# Jordanian views regarding sharing of medical data for research: A cross-sectional study during COVID-19 pandemic

**DOI:** 10.1371/journal.pone.0265695

**Published:** 2022-03-21

**Authors:** Moawiah Khatatbeh, Lobna F. Gharaibeh, Omar F. Khabour, Rana K. Abu-Farha, Karem H. Alzoubi

**Affiliations:** 1 Department of Basic Medical Sciences, Faculty of Medicine, Yarmouk University, Irbid, Jordan; 2 Pharmacological and Diagnostic Research Center, Faculty of Pharmacy, Al-Ahliyya Amman University, Amman, Jordan; 3 Department of Medical Laboratory Sciences, Jordan University of Science and Technology, Irbid, Jordan; 4 Department of Clinical Pharmacy and Therapeutics, Faculty of Pharmacy, Applied Science Private University, Amman, Jordan; 5 Department of Pharmacy Practice and Pharmacotherapeutics, University of Sharjah, Sharjah, UAE; 6 Department of Clinical Pharmacy, Jordan University of Science and Technology, Irbid, Jordan; Seoul National University College of Medicine, REPUBLIC OF KOREA

## Abstract

**Purpose:**

In the current study, the views of Jordanian regarding sharing medical reports for research purposes were investigated during the COVID-19 pandemic. In addition, motivators and barriers regarding sharing of medical records were examined.

**Methods:**

This observational survey-based cross-sectional study was conducted using an electronic questionnaire during the COVID-19 pandemic (second half of 2020). The questionnaire link was disseminated through two social media platforms (WhatsApp and Facebook), targeting Jordanian adults (age >18 years).

**Results:**

In this study, 1,194 participants agreed to complete the study survey. Results showed that 58.3% of them (n = 696) reported to be willing to share their medical data. while 17.6% of the participants (n = 210) showed hesitancy to share their medical information. The most important motivators as perceived by the study participants were helping other patients who have similar health conditions (n = 995, 83.3%). Moreover, fearing from stigma (n = 753, 63.1%), and the lack of confidence in data security and privacy (n = 728, 61.0%) were among the main barriers preventing participants from sharing their information. Finally, results showed that participants with higher educational level (bachelor or higher) (OR = 0.299, P<0.001), or those living in center of Jordan (OR = 0.270, P<0.001) showed a lower tendency to share their medical data. While participants those who have shared data before showed a higher tendency to share their medical data (OR = 2.524, P<0.001).

**Conclusion:**

In this study, many of the participants had a positive attitude towards sharing biomedical data for scientific research during the COVID-19 pandemic, many had doubts in the control over their data. Thus, policymakers and data users should address the concerns and values of patients and understand their preferences in favor of an ethically scrupulous use of data in research.

## 1. Introduction

There is a trend toward the use of the patients’ medical records for research purposes in developed countries such as the United Kingdom and the United States [1–4]. This is facilitated by the development of many ways to extract and process information in medical records with maintaining the personal information of patients unidentified [1]. Sharing of medical records continues to lead to an exciting range of health-related discoveries, improving population health and saving lives [1]. During pandemics such as the current COVID-19, rapid data sharing regarding infection duration, method of transmission, severity, age groups at risk, comorbidities, clinical data and management can maximize the utility of data and seems especially urgent and warranted [5], and becomes a moral obligation to save lives and provide solutions to end the pandemic[6, 7].

Several studies have investigated public views regarding sharing of medical records [8–12]. For example, studies conducted in the USA have found an overall positive orientation to the use of patient data for societal benefit [9–12]. However, a study that was conducted as part of the National Health Service England’s ill-fated Care data scheme indicated that certain schemes for secondary data use can prove unpopular in the UK and a reluctance of the public to share their medical records and to accept future attempts at extracting and linking large datasets of medical information [8]. This could be due to the risk that personal information can be identified and misused because of the sharing of medical records, which might cause harm to the patients [13].

Several ways have been suggested to reduce the risk that an individual patient could be re-identified. These include 1) removing the pseudonymization code and aggregating the data to a level at which re-identification is not possible. 2) Protecting patient sensitivity data with computing security systems, which do not allow the data to be downloaded. 3) Trusted and trained users are permitted access to the data [1]. Thus, a balance can be achieved to make sure patient privacy is protected and the societal benefit of medical research using patient data is achieved even during the COVID-19 era and other pandemics [14].

While the view of the public regarding sharing of medical data for research purposes was well investigated in developed countries [2, 4, 8, 10–12], however, public views and understanding about this work, has been lagging in the majority of developing countries. In Jordan, a study that was conducted on researchers showed that about 50% of them were positive about sharing biomedical data [15]. The same study reported that the lack of regulations and support for data deposition are barriers limiting data sharing in Jordan [15]. Another study from Jordan showed that data sharing practices of healthcare practitioners/researchers were not satisfactory in terms of confidentiality and data protection measures [16]. The previous two studies were conducted from Jordan on researchers/health care professionals [15, 16]. However, none of the studies examined public views in Jordan regarding the sharing of biomedical data for research purposes, especially during the COVID-19 pandemic. Therefore, in the current study, the views of Jordanian regarding sharing medical reports for research purposes were investigated during the COVID-19 pandemic. In addition, motivators and barriers regarding sharing of medical records were examined.

## 2. Methods

### 2.1 Study design and subjects

This observational survey-based cross-sectional study was conducted using an electronic questionnaire during the COVID-19 pandemic (second half of 2020). During the study period, a convenience sample of Jordanian adults (age >18 years) were invited to participate in this study to assess their views regarding sharing medical reports for research purposes during the COVID-19 pandemic. The study questionnaire was uploaded on Google form platform, then the questionnaire link was disseminated through two social media platforms (WhatsApp and Facebook). The questionnaire link was posted to several Jordanian pages to ensure the dives ensure the diversity of the recruited participants. Before filling the questionnaire, participants were informed about the purpose of the study, and that their participation is voluntary. Electronic informed consent was obtained before filling the survey, and the anonymity of respondents was preserved, as no information about their identity was collected.

### 2.2 Sample size calculation

The standard formula: n = P × (1- P) × z^2^/d^2^ was used to calculate a minimal sample size for this study. We determined the sample size based on the most conservative proportion (P = 50%), and using 5% desired precision, and confidence levels of 95% were used. A sample size of 385 were considered the minimum sample size needed form this study.

### 2.3 Questionnaire development and validation

The study questionnaire was developed by the research team following an extensive literature review of relevant studies [17–22]. It consisted of several closed-ended questions that were divided into four main sections. The first section was dedicated to retrieving participants’ sociodemographics such as age, gender, marital status, region of residence, education, and having chronic diseases. The second section evaluated participants’ willingness to share their medical data for research, participants were asked to select one out of three responses “Yes I am willing to share data, unsure, or no I am not willing to share my daya”. The latter section assessed participant’ perceptions towards motivators of sharing medical data. This section contains 11 statements to assess patricpants perceptions to differents motivators. The last section evaluated their perceptions towards the barriers of sharing medical data using 17 statements. The last two sections were assessed using a 3-point Likert scale (1 = disagree, 2 = neutral, and 3 = agree).

The final draft of the questionnaire was reviewed by a group of academics for face and content validity to evaluate its relevance, specificity, and comprehensiveness. ‎Consequently, some statements were remodeled based on their feedback. Afterward, the questionnaire ‎was translated to Arabic, and back-translated to English, and then, the translated version was compared with the original one ‎for validation. Also, the ‎internal reliability was tested using Cronbach’s alpha measure, which yielded 0.82, indicating that the ‎scale has an acceptable internal consistency.

### 2.4 Ethical considerations

The study was approved by the Instituational Review Board at Jordan University of Science and Technology (Reference number 24/132/2020). The study was conducted following the standards issued by the World Medical Association’s Declaration of Helsinki guidance [23].

### 2.5 Statistical analysis

Obtained outcomes were entered and analyzed using IBM statistical package for social sciences (IBM SPSS Statistics, version 22.0, Chicago, Illinois). Descriptive analyses were presented as median ± interquartile range (IQR) for continuous variables, while frequency and proportions were used for categorical variables.

Univariate linear regression analysis was performed to screen predictors affecting public willingness to share their medical data, and all variables with P-value< 0.25 were entered into multiple linear regression analysis. Variables that independently affected affecting public willingness to share their medical data were identified in the multiple linear regression analysis. Variables independence was checked using person correlation where <0.9 indicates the absence of multicollinearity between the independent variables in regression analysis. A P-value of ≤0.05 was considered statistically significant. Cronbach’s α was used to evaluate the reliability of the questionnaire i.e. that the scales constructed are fit for their purpose, with values ≥ 0.7 indicating acceptable internal consistency [24].

## 3. Results

During the study period, 1,194 responses were received. Around 39% of the participants aged between 18–24 years (n = 470, 39.4%), and about 84.1% of them (n = 1004) had a bachelor degree of higher. Moreover, around one-third of the participants were married (n = 393, 32.9%), and around two-third of them live in the north of Jordan (n = 792, 66.5%). Only 17.1% of the participants (n = 204) had a chronic medical conditions, and around 35% of them (n = 416, 34.8%) had shared their medical data before. For more details about the socio-demographic and medical information, refer to **Table 1**.

**Table 1:** Socio-demographic characteristics of the study population (n = 1194).

Characteristic	n (%)
Gender • Male • Female	652 (54.6) 542 (45.4)
Age (year) • 18–24 • 25–34 • 35–44 • ≥45	470 (39.4) 455 (38.1) 169 (14.2) 100 (8.4)
Marital status • Single • Married • Divorced/Widow	794 (66.5) 393 (32.9) 7 (0.6)
Region of residence • North of Jordan • Central of Jordan • South of Jordan	792 (66.3) 351 (29.4) 51 (4.3)
Level of education • High school or below • Bachelor degree • Postgraduate	190 (15.9) 904 (75.7) 100 (8.4)
Have chronic diseases • No • Yes	990 (82.9) 204 (17.1)
Shared data before • No • Yes	778 (65.2) 416 (34.8)

**Fig 1:**
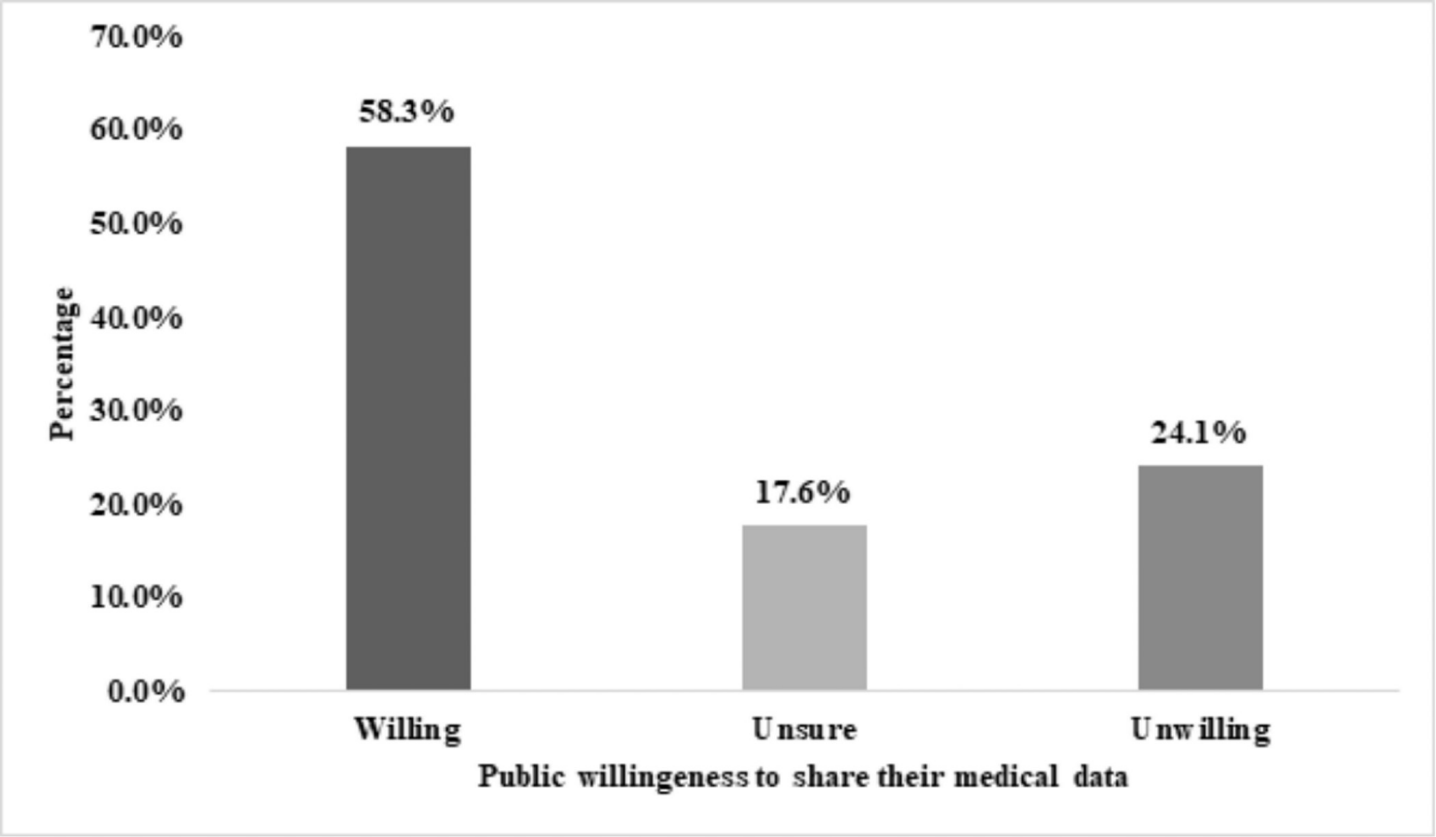
Public willingness to share their medical data (n = 1194).

Participants willingness to share their medical data during the COVID-19 pandemic are presented in **Fig 1.** Results showed that 58.3% of them (n = 696) reported to be willing to share their data, while 24.1% of them (n = 288) refused to do so. Moreover, 17.6% of the participants (n = 210) showed hesitancy to share their medical information.

Participants perception towards motivators to share their medical data were assessed and presented in **Table 2**. The most important motivators as perceived by the study participants were helping other patients who have similar health conditions (n = 995, 83.3%), if their social security number and telephone number are not gathered (n = 903, 75.2%), and knowing how data will be protected (n = 893, 74.8%). Financial benefits were the least reported motivator to share medical data as reported by the participants (n = 532, 44.6%).

**Table 2:** Public perceptions towards medical data sharing motivators (n = 1194).

In your opinion, what are conditions motivate you to share health data?	n (%)
If I’m able to know how my data will be protected	893 (74.8)
If my social security number and telephone number are not gathered	903 (75.2)
If my social security number and telephone number, if collected, are removed from the data	835 (69.9)
When data sharing could lead to better patient care through improved diagnosis and treatment	830 (69.5)
If data sharing is paid (financial benefits)	532 (44.6)
If I feel that sharing is for the common good	574 (48.1)
If data sharing is with public (governmental) companies	623 (52.2)
I feel that the benefits of sharing healthcare data outweigh the risks	614 (51.4)
If I trust the researcher or if I know him personally	767 (64.2)
I tend to support of research in general	797 (66.8)
To help other patients who have similar conditions to my health problem	995 (83.3)

**Table 3** shows participants’ perceptions of data sharing barriers. The most important barriers preventing participants from sharing their information were feeling that their data may lead to stigma (n = 753, 63.1%), the lack of confidence in data security and privacy (n = 728, 61.0%), having concerns about potential misuse by insurers, the government and other third parties (n = 697, 58.4%), and feeling not comfortable with researchers accessing their data (n = 691, 57.9%). For more information about the perceived barriers, refer to **Table 3**.

**Table 3:** Public perceptions towards medical data sharing barriers (n = 1194).

Statements	n (%)
If I feel not comfortable with researchers accessing my data	691 (57.9)
If I feel that my health data is being re-identified and disclosed to people who I know	491 (41.1)
If the purpose of the research is hazy/unclear	615 (51.5)
If data sharing is with private companies	565 (47.3)
If I have concerns about misuse of data	574 (48.1)
Lack of confidence in data security and privacy	728 (61.0)
Concerns about potential for data to be sold on to other organizations and used for purposes other than research	682 (57.1)
Concerns about potential misuse by insurers, the government and other third parties	697 (58.4)
Concerns on transparency about how data are used and how it might be used in the future	688 (57.6)
Share non-routine data	641 (53.7)
If data collection process takes a long time	536 (44.9)
Lack of trust in the research team	549 (46.0)
If my health data may lead to stigma (e.g: STDs, Scabies)	753 (63.1)
If my health data is being disclosed to researchers or doctors not involved in my care	563 (47.2)
Concerns about issues related to decision making and who decides who gets access to data and who does not	626 (52.4)
If I have concerns about data management plans	548 (45.9)
If data collection forms/tools include unclear language or terminology	598 (50.1)

Finally, factors affecting public willingness to share their medical data were investigated using univariate and multivariate linear regression analysis (**Table 4**). Results showed that participants with higher educational level (bachelor or higher) (OR = 0.299, P<0.001) or those living in center of Jordan (OR = 0.270, P<0.001) showed a lower tendency to share their medical data. While participants those who have shared data before showed a higher tendency to share their medical data (OR = 2.524, P<0.001).

**Table 4:** Assessment of predictors affecting public willingness to share their medical data (n = 1194).

Parameter	Willingness to share data [0: No/unsure, 1: Yes]			
	OR	P-value#	OR	P-value$
Gender • Male • Female	Reference 0.771	0.057^	0.836	0.316
Age (year)	0.998	0.623	----	----
Marital status • Married • Others (single, divorced, widowed)	Reference 0.881	0.373	----	----
Region of residence • North of Jordan • Central of Jordan • South of Jordan	Reference 0.253 1.694	<0.001^ 0.090^	0.270 1.694	<0.001* 0.091
Level of education • High school or below • Bachelor degree or higher	Reference 0.346	<0.001^	0.299	<0.001*
Have chronic diseases • No • Yes	Reference 0.885	0.506	----	----
Shared data before • No • Yes	Reference 2.106	<0.001^	2.524	<0.001*

# using simple logistic regression

$ using multiple logistic regression

^ eligible for entry in multiple logistic regression

* significant at 0.05 significance level.

## 4. Discussion

Sharing of biomedical data has been shown to improve population health and save lives [1], especially during health crises such as the current COVID-19 pandemic [6, 7]. Despite the tremendous amount of research done on the public´s opinion regarding sharing of medical information, the issue of who owns the data is still controversial and there is no consensus on who is the actual owner of that data [25]. Medical institutions regard the patient’s medical information as their own, while in fact, they are data collectors and keepers. Based on that fact, patients own their medical data and its use for research purposes should address their attitudes and concerns [26]. Some patients are very suspicious about the use of their data for any purpose. Their skepticism regarding the use of data for commercial reasons, true benefits of data sharing, and security issues may hinder the use of data in research [27].

Our study showed that only 58% of patients were willing to share data during the COVID-19 pandemic and 17.6% showed hesitancy. This percentage is lower than that of 78.8% detected in a study conducted in Canada [22]. Another study, that was conducted in the United States with a population that was older than in our study, showed that 74.8% were willing to share their data for research [28]. This lower percentage may be caused by cultural differences or simply disparities in the population of the studies.

Sharing medical data for research purposes is not a trivial issue for the public. Concerns of wavering their privacy rights for the progress of research and general knowledge are valid and legitimate. People support the use of their information for research purposes more than utilizing it for quality improvement aspects in medical institutions for the hope of providing benefits to patients [4].

Similar to other studies, our results showed that one of the barriers was a lack of confidentiality. The concerns people had for approving the use of their data included lack of confidence in data security and possible abuse of data were also echoed in other studies [20]. Aitken et al conducted a systematic review that included 25 studies and examined public perspectives towards sharing health data for research. The review revealed similar obstacles and concerns among which are: confidentiality, oversight over data, and possible abuse of data [1].

Anonymity is an important factor that encourages people to share their data. People in our study were motivated to share their information if their security number and telephone number were not gathered (75.2%), which gives them a sense of security and privacy. The use of de-identified data for research purposes was a strong motive for participants in the United Kingdom health service [29]. Weitzman et al revealed that 90% of people enrolled in their study were willing to share their data for research if rigorous confidentiality measures were implemented [12]. These views concerning de-identifying personal information to ensure data security were reflected in many studies, which suggests the importance of this strategy to guarantee confidentiality and privacy [22, 30].

Geographical distribution and educational level affected willingness to share data, where people in central areas of Jordan, and those with higher education were less willing to share their electronic records information compared to those living in the northern areas of Jordan. This can be explained by educational and social differences that can impact that decision. Ethnic background, although is not the same as geographical distribution, was extensively studied as a factor that might affect the tendency to share data for research, but the available evidence is controversial [28, 31]. In addition, people who have shared data before may be more willing to share their information for research for the hope of medical advancement, providing better health care, benefitting them and other patients.

The strength of this study is that, to our knowledge, this is the first study conducted to explore attitudes of the public towards data sharing in Jordan. The study was conducted in the COVID-19 pandemic, which reflects the attitudes of individuals during this stressful time where data sharing is crucial for public health and global general knowledge. This also allows for the possibility of comparison between the results of this study and future studies after the pandemic is over.

The study has some limitations. The questionnaire was distributed through social media platforms, people without access to these applications or nonusers would not be able to access this questionnaire. Filling questionnaires without interviews might be susceptible to misunderstandings that cannot be explained to the participant. In addition, most of the participants in our study were younger than 35 years of age, including older participants might lead to altered results. Finally, the study was conducted during the COVID-19 pandemic and thus, the Jordanian views regarding sharing medical data for research might vary in ordinal situations.

Findings from this study shed the light on possible inclinations and concerns of individuals towards access to their health information. Institutions and entities interested in research must address these issues to promote the acceptance of data sharing among the public. Enhancing the acceptance of data sharing will benefit medical research and contribute to representation of Jordan and other developing countries scientific information to health care that is a global burden.

## 5. Conclusion

The enormous increase in data gathered in electronic health records provides an enormous source of valuable information for research. Although more than half of the Participants were willing to share their data for scientific research during the COVID-19 pandemic, many barriers were identified by the participants such as potential misuse by insurers and being uncomfortable with researchers accessing their data. Having chronic diseases and higher educational levels led to lower tendencies towards data sharing. To enhance positive attitudes and willingness of the public towards data sharing, policymakers, legislators, and data users should address barriers and understand public preferences in favor of an ethically scrupulous use of data in research.
